# Microsurgical resection of an intravestibular schwannoma: a review of surgical technique and management considerations

**DOI:** 10.3171/2021.7.FOCVID2187

**Published:** 2021-10-01

**Authors:** Ramin A. Morshed, Alexander F. Haddad, Kunal P. Raygor, Mary Jue Xu, Charles J. Limb, Philip V. Theodosopoulos

**Affiliations:** Departments of 1 Neurological Surgery and; 2 Otolaryngology–Head and Neck Surgery, University of California, San Francisco, California

**Keywords:** microsurgery, intravestibular, schwannoma, translabyrinthine

## Abstract

Intravestibular schwannomas are rare tumors within the intralabyrinthine region and involve different management considerations compared to more common vestibular schwannomas. In this report, the authors review a case of a 52-year-old woman who presented with hearing loss and vestibular symptoms and was found to have a left intravestibular schwannoma. Given her debilitating vestibular symptoms, she underwent microsurgical resection. In this video, the authors review the relevant anatomy, surgical technique, and management considerations in these patients.

The video can be found here: https://stream.cadmore.media/r10.3171/2021.7.FOCVID2187

## Transcript

In this surgical video, we describe the management of a patient found to have an intravestibular schwannoma and review surgical technique as well as relevant anatomy.

### 0:30 Patient Information

The patient is a 52-year-old female who presented with profound left-sided hearing loss, tinnitus, ear fullness, and imbalance leading to falls. Other past medical history included a distant diagnosis of colon cancer which had been resected and hyperthyroidism.

### 0:46 Physical Examination

Her physical examination revealed significant left-sided hearing loss, but she was otherwise neurologically intact. Otomicroscopy performed bilaterally was unrevealing.

### 0:56 Audiogram

Audiogram results demonstrated sensorineural hearing loss on the left side with no response at 90 dB and 0% word recognition. Right-sided hearing was preserved.

### 1:08 MRI

An MRI was obtained demonstrating a small mass within the left vestibule projecting into the horizontal and superior semicircular canals concerning for an intravestibular schwannoma, as demonstrated on these T1 postcontrast and FIESTA sequences. The mass did not extend into the internal auditory canal or cochlea.

### 1:29 Management Options

Management options for intravestibular schwannomas can include microsurgical resection, radiosurgery, and conservative management with serial imaging. Given the patient’s hearing loss and ongoing debilitating vestibular symptoms, microsurgical resection of the mass was recommended via a translabyrinthine approach. Recent meta-analyses in the literature suggest that the majority of patients treated with microsurgery will experience improvement in their vestibular symptoms on follow-up. If surgery is pursued, cochlear implant placement can be performed at the time of resection. As there is a risk of cochlear ossification on follow-up which can prevent cochlear implant placement, our group typically proceeds with placement at the time of tumor removal or within 3–6 months prior to ossification. In this case, consideration was given to placing a cochlear implant at the time of surgery, but the patient elected for tumor resection only.

### 2:27 Surgical Procedure

She subsequently was taken to the OR for tumor resection. The patient was placed in the supine position with the head turned to the right. Facial nerve monitoring was utilized for intraoperative localization and preservation.

**2:41** A semicircular postauricular incision was made down to the temporalis fascia. The mastoid periosteum was incised and elevated. Next, a mastoidectomy was performed. The anterior, posterior, and inferior borders of this presigmoid approach correspond to the external ear canal, the sigmoid sinus, and the mastoid tip, respectively.

**3:02** The microscope was then brought into the operating field. Using a cutting burr, a mastoidectomy was performed. Drilling proceeded through the air cells of the mastoid bone until the antrum was visualized. During this approach, the bone overlying the tegmen, sigmoid sinus, and ear canal was kept intact.

**3:21** A diamond burr was then used to define the horizontal canal and open the antrum wider to define the incus, which can be seen partially exposed within the antrum in this operative video.

**3:35** Using the diamond burr, air cells were further removed until the dense bone of the horizontal canal was visualized and blue-lined.

**3:46** At this portion of the case, the horizontal canal can be seen blue-lined. Drilling proceeds along the center of this line or superior to it to avoid injury to the facial nerve which is located inferiorly.

**4:02** Once the facial recess had been defined, the posterior semicircular canal was drilled using a diamond burr. Here, the posterior canal has been blue-lined.

**4:14** The superior semicircular canal was also further defined at this point in the case. The horizontal semicircular canal was then entered, making sure to avoid injury to the facial nerve located inferior and slightly lateral.

**4:30** The tumor was noted along the anterior limb of the horizontal canal. This was followed into the vestibule.

**4:48** Once the tumor was exposed, microinstruments were used to dissect the tumor out of the vestibule.

**5:11** Here, the component of the tumor extending into the superior semicircular canal was dissected and pulled down toward the main component within the vestibule.

**5:30** During this time, the facial nerve with overlying bone stimulated at 0.5 mA.

**5:43** A gross-total removal of the mass was performed without changes in facial nerve stimulation threshold.

**6:01** For reconstruction, a bone pate was placed over the vestibule and exposed semicircular canals. A fat graft was not used to simplify approaches to this region if cochlear implant placement was considered in the future. There was no CSF leak during the surgery. The rest of the closure was performed in layers in a typical fashion. There were no complications.

### 6:24 Pathology

Pathology demonstrated a WHO grade I schwannoma. Postoperatively, the patient had no facial weakness. Her vestibular symptoms improved shortly after the surgery, and the patient is currently undergoing planning for a left cochlear implant.

**6:40** Intralabyrinthine schwannomas are rare tumors of the vestibular or cochlear nerves that can result in hearing loss, vertigo, and tinnitus, often presenting with Ménière’s-like symptomatology. Typically, high-resolution images of the affected temporal bone can be obtained to help with diagnosis and tumor localization. Prior studies have categorized these tumors based on specific location and involvement of either the cochlea or vestibule and based on presence of extension into the internal auditory canal.

**7:10** Here are tumor locations within the labyrinth that have been previously defined. Intravestibular lesions may or may not involve extension into the semicircular canals.

### 7:20 Conclusions

Hearing preservation is not possible with surgical approaches to these tumors, and thus intervention is often reserved for patients with nonserviceable hearing and growth on imaging. Additional surgical indications include intractable vestibular symptoms, which often respond well to intervention. Radiosurgery may be another treatment option but may exacerbate hearing loss. Furthermore, it is unclear of the impact of this treatment modality on long-term vestibular outcomes. Overall, evidence from the literature suggests that symptoms such as vertigo improve in the vast majority of patients after microsurgical resection and remain stable for patients treated with SRS.

### 8:02 References

^1–5^

## Disclosures

Dr. Limb reports being a consultant for and receiving research support from Advanced Bionics Corporation, Med-El Corporation, and Oticon Medical; and being the chief medical officer of and a stock shareholder in Spiral Therapeutics.

## Author Contributions

Primary surgeon: Theodosopoulos, Limb. Assistant surgeon: Xu. Editing and drafting the video and abstract: Theodosopoulos, Morshed, Haddad, Raygor. Critically revising the work: Theodosopoulos, Morshed, Haddad, Raygor, Limb. Reviewed submitted version of the work: all authors. Approved the final version of the work on behalf of all authors: Theodosopoulos. Supervision: Morshed.
